# Integral membrane proteins and free electron lasers – a compatible couple indeed!

**DOI:** 10.1107/S2052252515012294

**Published:** 2015-06-30

**Authors:** Michael C. Wiener

**Affiliations:** a Department of Molecular Physiology and Biological Physics, University of Virginia, Charlottesville, VA 22908-0886, USA

**Keywords:** XFEL, P-type ATPases, ligand screening, serial femtosecond crystallography

## Abstract

Several structures of membrane transport proteins in complex with mechanistically-relevant ligands, determined by serial femtosecond crystallography of microcrystals at an X-ray free-electron source source, are presented. These results, including investigation of approaches to data quality assessment and refinement from low-redundancy data, indicate the feasibility of using this approach for ligand screening.

The first integral membrane protein X-ray crystal structure was solved in 1985 (Deisenhofer *et al.*, 1985 ▸). Thirty years later, they remain a frontier of structural biology, and an area of intense fundamental and practical interest. The structural and functional bases of many critical biological processes that occur in and across membranes remain largely unknown. And, on the practical side, membrane proteins are considered to be the target of many, if not most, current and future drugs (Yildirim *et al.*, 2007 ▸). Yet, they are statistically highly underrepresented in the PDB, with only 541 unique structures in the ‘Membrane Proteins of Known Structure Database’ (http://blanco.biomol.uci.edu/mpstruc/). Why the dearth of structures of such important and fascinating macromolecules? Multiple nontrivial technical obstacles exist (Wiener, 2004 ▸). Production of ‘crystallization’ quantities of purified stable membrane proteins, particularly of eukaryotic proteins, is frequently time- and cost-prohibitive. Membrane protein production requires solubilization, by detergents, of the membrane in which the protein is situated, and replacement of this membrane by a membrane mimetic, virtually always a detergent or other amphipathic molecule. Therefore, the entity that goes into crystallization screening is a protein–detergent complex (PDC), where the hydrophobic membrane-spanning surface of the protein is shielded by a torus of detergent. Crystallization approaches common to soluble proteins, such as vapor diffusion, batch crystallization, and free-interface diffusion, are often used; however, the commercial and in-house crystallization screens used are often different from those used for soluble proteins. Given the importance of the membrane for membrane protein function and stability, dramatic success has been achieved with methods that present a more membrane-like local environment. These include, most prominently, the lipidic cubic phase (Landau & Rosenbusch, 1996 ▸) and bicelles (Faham & Bowie, 2002 ▸). Membrane protein crystals, though often relatively straightforward to obtain, are high solvent content entities that all too often diffract weakly and to low resolution.

Enter the X-ray free electron laser (XFEL), with an instantaneous brightness ~10^9^ greater than synchrotron sources (Chapman, 2009 ▸). Protein crystals are radiation sensitive, with 20–30 MGy, referred to as the Henderson–Garman limit, the deposited dose at which ~50% loss of diffraction intensity is observed (Henderson, 1990 ▸; Owen *et al.*, 2006 ▸). [The value of this limit can be much lower for metalloproteins (Yano *et al.*, 2006 ▸; Hough *et al.*, 2008 ▸).] With such limits, what is the utility of a tremendously more intense X-ray source? The femtosecond time-signature of the XFEL is what makes these sources a truly transformative technology. In 2000, results of computer simulation led Neutze *et al.* to predict that, with sufficiently short pulse lengths of a few tens of femtoseconds or less, diffraction would occur faster than radiation damage (Neutze *et al.*, 2000 ▸). This ‘diffract before destroy’ hypothesis made a bold claim, which was subsequently realised in 2011, when Bragg peaks were observed from sub-micron crystals of Photosystem II (PS II) delivered to an XFEL source (Chapman *et al.*, 2011 ▸). Rapid and spectacular success has been reported in multiple systems, including G-protein coupled receptors, an integral membrane protein class of intense biomedical/pharmaceutical interest (Liu *et al.*, 2014 ▸). Many in the macromolecular crystallography community are wondering whether SFX will be able to be used (relatively) routinely in structure determination. How challenging is it to produce micro- and nanocrystals suitable for SFX structure determination? And, for applications such as structure-based drug discovery, where tens or hundreds of structures of lead-compound/target complexes need to be solved in quick succession, will SFX provide this capability for ‘difficult’ targets such as integral membrane proteins? Some of these concerns arise from inherent differences between SFX and synchrotron data collection. Substantial pulse-to-pulse intensity variation currently exists at XFEL sources, making experimental phase determination at present a risky endeavor. Also, current SFX sample injection and mounting methods yield one room-temperature diffraction pattern, consisting entirely of partial reflections, for each randomly oriented crystal that successfully intersects the beam. Thus, Monte Carlo integration methods (Kirian *et al.*, 2010 ▸), rather than standard X-ray crystallographic data reduction approaches, are used, and very low crystal ‘hit rates’ are often reported.

Bublitz *et al.* (2015 ▸) present results that are a significant step towards demonstration of feasibility of SFX as a general method for structure determination, at least by molecular replacement. Their structural targets are P-type ATPases, ubiquitous transporters functioning as ion and lipid pumps, found in all kingdoms of life. P-type ATPases in pathogens may be viable drug targets, and some indication of a role for a human ortholog in cancer has been reported (mentioned in the paper). Their results indicate that microcrystals may be relatively straightforward to obtain, although, like all other macromolecular crystallization, systematic screening is required. Also, they determine structures of three complexes of SERCA, a mammalian P-type ATPase, with various mechanistically relevant small molecules bound. They apply recently described methods of macromolecular crystallographic data analysis (Karplus & Diederichs, 2012 ▸) to accurately characterize the resolution of their data. After obtaining solutions by molecular replacement, a clever analysis is performed by comparing *R*_free_ *versus* resolution for collected datasets *versus* ‘scrambled’ datasets, where structure factor amplitudes are transposed to ‘incorrect’ indices. Strikingly, these plots show a clear increase in *R*_free_ in the ‘scrambled’ resolution range, showing that there is nonzero information content in data that many might consider too inaccurate to be of utility in structure determination. Lastly, as another demonstration that modest-resolution XFEL data, even of low-redundancy and completeness, can be useful, the authors locate bound ligands in *F_o_ − F_c_* and anomalous difference Fourier maps. These are very exciting times in structural biology, where XFEL sources and new/next-generation microfocus synchrotron beamlines, along with paradigm-shifting technological advances in electron microscopy (Liao *et al.*, 2013 ▸) and electron diffraction (Shi *et al.*, 2013 ▸), truly promise to deliver more from less. Even with these methods, however, the stringent requirements of sample preparation will ultimately be the rate- and resolution-limiting step.
